# Happy emotion cognition of bimodal audiovisual stimuli optimizes the performance of the P300 speller

**DOI:** 10.1002/brb3.1479

**Published:** 2019-11-15

**Authors:** Zhaohua Lu, Qi Li, Ning Gao, Jingjing Yang, Ou Bai

**Affiliations:** ^1^ School of Computer Science and Technology Changchun University of Science and Technology Changchun China; ^2^ Department of Electrical and Computer Engineering Florida International University Miami FL USA

**Keywords:** audiovisual, brain–computer interface, emotional cognition, face, P300 speller, voice

## Abstract

**Objective:**

Prior studies of emotional cognition have found that emotion‐based bimodal face and voice stimuli can elicit larger event‐related potential (ERP) amplitudes and enhance neural responses compared with visual‐only emotional face stimuli. Recent studies on brain–computer interface have shown that emotional face stimuli have significantly improved the performance of the traditional P300 speller system, but its performance needs to be further improved for practical applications. Therefore, we herein propose a novel audiovisual P300 speller based on bimodal emotional cognition to further improve the performance of the P300 system.

**Methods:**

The audiovisual P300 speller we proposed is based on happy emotions, with visual and auditory stimuli that consist of several pairs of smiling faces and audible chuckles (E‐AV spelling paradigm) of different ages and sexes. The control paradigm was the visual‐only emotional face P300 speller (E‐V spelling paradigm).

**Results:**

We compared the ERP amplitudes, accuracy, and raw bit rate between the E‐AV and E‐V spelling paradigms. The target stimuli elicited significantly increased P300 amplitudes (*p* < .05) and P600 amplitudes (*p* < .05) in the E‐AV spelling paradigm compared with those in the E‐V paradigm. The E‐AV spelling paradigm also significantly improved the spelling accuracy and the raw bit rate compared with those in the E‐V paradigm at one superposition (*p* < .05) and at two superpositions (*p* < .05).

**Significance:**

The proposed emotion‐based audiovisual spelling paradigm not only significantly improves the performance of the P300 speller, but also provides a basis for the development of various bimodal P300 speller systems, which is a step forward in the clinical application of brain–computer interfaces.

## Graphical abstract

We designed a new audiovisual P300 speller system based on the congruence of happy emotions in faces and voices to verify whether bimodal emotional face and voice stimuli can further improve the spelling accuracy of such a system, compared with visual‐only face stimuli. We assessed the validity of the hypothesis by analyzing the event‐related potentials elicited by the target stimuli and comparing the spelling accuracy between the bimodal and unimodal paradigms.

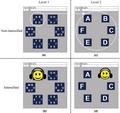

## 1 INTRODUCTION

Brain–computer interface (BCI) systems offer a new direct communication channel between the brain and the outside world for patients with severe neuromuscular disease, such as amyotrophic lateral sclerosis or progressive muscular dystrophy, who have normal cognitive function (Carelli et al., 2017; Lazarou, Nikolopoulos, Petrantonakis, Kompatsiaris, & Tsolaki, 2018; Wolpaw, Birbaumer, McFarland, Pfurtscheller, & Vaughan, 2002). BCI systems usually translate the intentions of a user into computer commands by noninvasively recording electroencephalography (EEG) signals on the head surface (Allison, Wolpaw, & Wolpaw, 2007; Daly & Huggins, 2015; Monge‐Pereira et al., 2017).

The P300, a classical EEG signal, is an event‐related potential (ERP) elicited by the oddball event that occurs about 300 ms after stimulus onset (Bernat, Shevrin, & Snodgrass, 2001). The first P300‐based spelling system was introduced by Farwell and Donchin (1988), who developed a classical row/column flashing spelling paradigm in which 26 letters and 10 numbers are arranged into a 6 × 6 matrix. If a user wants to output a character (i.e., the target character), he/she only needs to focus on the target character and ignore other characters; as the flashing probability of a row/column containing the target character is 1/6, this represents an oddball event and P300 potentials are thus elicited. The target character is then outputted via signal classification methods. This system enables direct communication between the brain and the outside world; however, the P300 speller system is not yet considered satisfactory due to its low and unstable accuracy (Rezeika et al., 2018).

Subsequently, a number of studies have been conducted to improve the performance of P300 speller systems, which improved the spelling accuracy in mainly two different ways: by optimizing the system's detection methods of EEG signals (Blankertz, Lemm, Treder, Haufe, & Muller, 2011; Krusienski et al., 2006; Li, Shi, Gao, Li, & Bai, 2018) and by designing new spelling paradigms (Li, Lu, Gao, & Yang, 2019; Pires, Nunes, & Castelo‐Branco, 2012; Townsend et al., 2010). One hot topic in the optimization of P300 spelling paradigms is to increase their reliability and accuracy by detecting larger‐amplitude ERPs or more component ERPs elicited by new spelling paradigms. For example, Kaufmann, Schulz, Grunzinger, and Kubler (2011) proposed the famous face P300 spelling paradigm, in which the character was intensified by covering it with a famous face; this new paradigm improved the performance of P300 speller systems by eliciting larger‐amplitude P300 potentials and other obvious ERP components, such as N170 and N400. Based on these developments, some studies then proposed face spelling paradigms with faces that express emotions; the performance of P300 spelling paradigms could indeed be optimized using changes in emotion that reduce adjacent interference and fatigue (Jin, Daly, Zhang, Wang, & Cichocki, 2014). Chen, Jin, et al. (2016) later combined different colors and facial emotions to evoke higher P300 and N400 ERP amplitudes and further improve the classification accuracy of spelling paradigms.

The perception of emotions in our everyday life is often based on bimodal audiovisual information. Some studies have shown that the P300 is more sensitive to cross‐modal audiovisual than to unimodal visual emotion expressions. In a comparison of emotion‐based oddball paradigms with either audiovisual stimuli (such as a happy face with a happy voice or a sad face with a sad voice) or only a visual stimulus (such as a happy face or a sad face), the emotion‐based audiovisual stimuli elicited larger amplitudes and shorter latencies of the P300 than those elicited by the visual‐only face stimuli (Campanella et al., 2010). Chen, Han, Pan, Luo, and Wang (2016) reported that P300 amplitudes were larger for bimodal stimuli (face and voice with emotion) than for the sum of two unimodal stimuli (face stimulus and voice stimulus). Other studies also showed increased P300 amplitudes for audiovisual emotion stimuli (Chen, Pan, et al., 2016; Chen, Pan, Wang, Zhang, & Yuan, 2015). In an fMRI study on emotional voices and faces, the bilateral posterior superior temporal gyrus (pSTG) and the right thalamus showed enhanced activation and strength of the BOLD response during bimodal conditions (Kreifelts, Ethofer, Grodd, Erb, & Wildgruber, 2007). Behavioral data from the same study showed that audiovisual stimuli significantly increased the accuracy of stimuli detection compared with that of only visual or only auditory stimuli (Kreifelts et al., 2007).

In addition, when emotion expressions in the voice and face were congruent (such as a happy face combined with a happy song), a greater BOLD response was observed in the STG (Jeong et al., 2011). Congruent face and voice stimuli also resulted in faster response times, more accurate detection of stimuli, and enhanced brain activity (such as in the bilateral pSTG and the fusiform gyrus) compared with unimodal face stimuli (Collignon et al., 2008; Kreifelts et al., 2007). In particular, stimuli with congruent happy emotions in voices and faces enhanced the STG activation compared with stimuli with congruent sad emotions in voices and faces (Jeong et al., 2011). Compared with congruent neutral emotions in faces and voices, happy audiovisual stimuli also elicited more positive P200 and P300 amplitudes (Liu et al., 2012).

In this study, we propose a novel audiovisual P300 speller system based on the congruence of happy emotions in faces and voices (e.g., a smiling face presented with an audible chuckle) to further improve the performance of P300 spellers.

## 2 MATERIALS AND METHODS

### 2.1 Subjects

Nineteen healthy subjects (13 males) aged 22–28 (mean, 24.6 ± 2.13) years were recruited from the Changchun University of Science and Technology undergraduate and graduate participant pool. None of the subjects had vision or hearing impairments. The protocol was approved by the Ethics Committee of Changchun University of Science and Technology (CUST), and the study was performed in accordance with the recommendations of the committee. All subjects provided written informed consent prior to the experiment. All participants were native Chinese speakers, but were familiar with the Western characters used in the paradigm.

### 2.2 The spelling paradigms

**Figure 1:**
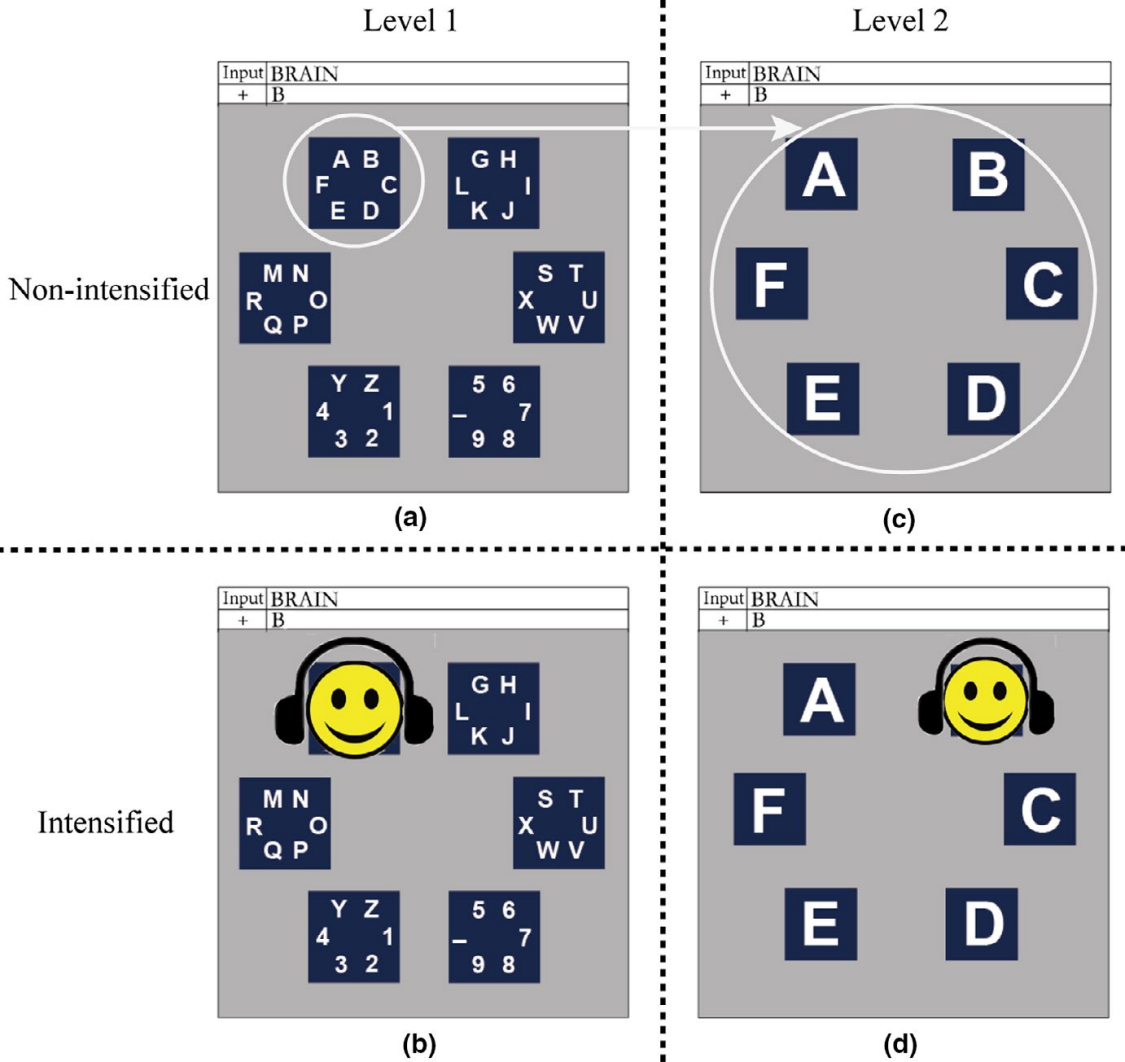
Experimental paradigm of the emotion‐based audiovisual (E‐AV) P300 speller. (a) Layout of level 1; (b) a sample for an intensified group‐area in level 1; (c) a sample for the layout of level 2 that corresponds to the top left group‐area in level 1; (d) a sample for an intensified subarea in level 2. The face photograph in the original setup was replaced by a smiling cartoon face in the figure, to avoid infringement of personal information

The proposed audiovisual P300 spelling paradigm (“E‐AV spelling paradigm”) is based on happy emotions and the traditional region flashing paradigms (Fazel‐Rezai, Gavett, Ahmad, Rabbi, & Schneider, 2011). The region flashing P300 spelling paradigm has two levels: level 1 consists of several group‐areas, each comprising several different characters, and level 2 consists of several subareas, each containing one character, with level 2 representing the spread of the group‐area in level 1. We arranged 36 characters into six group‐areas (Figure 1a, level 1). The six characters in each group‐area were arranged with a radius of 1.5 cm in a blue square (Takano, Komatsu, Hata, Nakajima, & Kansaku, 2009), and the six group‐areas were arranged with a radius of 5 cm on the screen. The layout of level 2 was similar to that of level 1 (Figure 1c), with six characters, each in a blue square, arranged with a radius of 5 cm on the screen. The visual and auditory stimuli were smiling faces and chuckles corresponding to the smiling faces. Because an earlier study on feature‐selective attention in audiovisual integration showed enhanced neural responses to human face and voice stimuli that differ in age and sex (Li et al., 2015), we selected six pairs of smiling faces and chuckles representing males and females in the three stages of childhood, youth, and old age. Our stimulus set thus comprised not only large visual differences in age and sex, but also large tone differences in age and sex. Each smiling face and chuckle pair corresponded to one group‐ or subarea; that is, a group‐area or a subarea was covered by the smiling face and the chuckle was presented via headset at the same time when the group‐area or subarea was intensified on the screen. For example, for the target character “B,” when the group‐area containing the target character (e.g., the top left group‐area) was intensified, this group‐area would be covered by a smiling face, and the chuckle corresponding to the smiling face was presented at the same time (Figure 1b). After a group‐area was selected, the screen display would transform to level 2; that is, subareas were displayed, representing the spread of the group‐area containing the target character. When the subarea containing the target character (e.g., the top right subarea) was intensified, this subarea would be covered by a smiling face, and the chuckle corresponding to the smiling face was presented at the same time (Figure 1d). The six group‐areas and subareas were all intensified in pseudorandom order with an interstimulus interval of 250 ms, with each group‐area/subarea intensified for 180 ms; the screen then reverted to the background for 70 ms.

As a control paradigm, we used the smiling face P300 spelling paradigm based on vision only (“E‐V spelling paradigm”), with the layout of levels 1 and 2 and the stimulus presentation identical to those of the E‐AV spelling paradigm, with the exception that no chuckle stimulus was presented.

### 2.3 Experiment procedure

**Figure 2:**
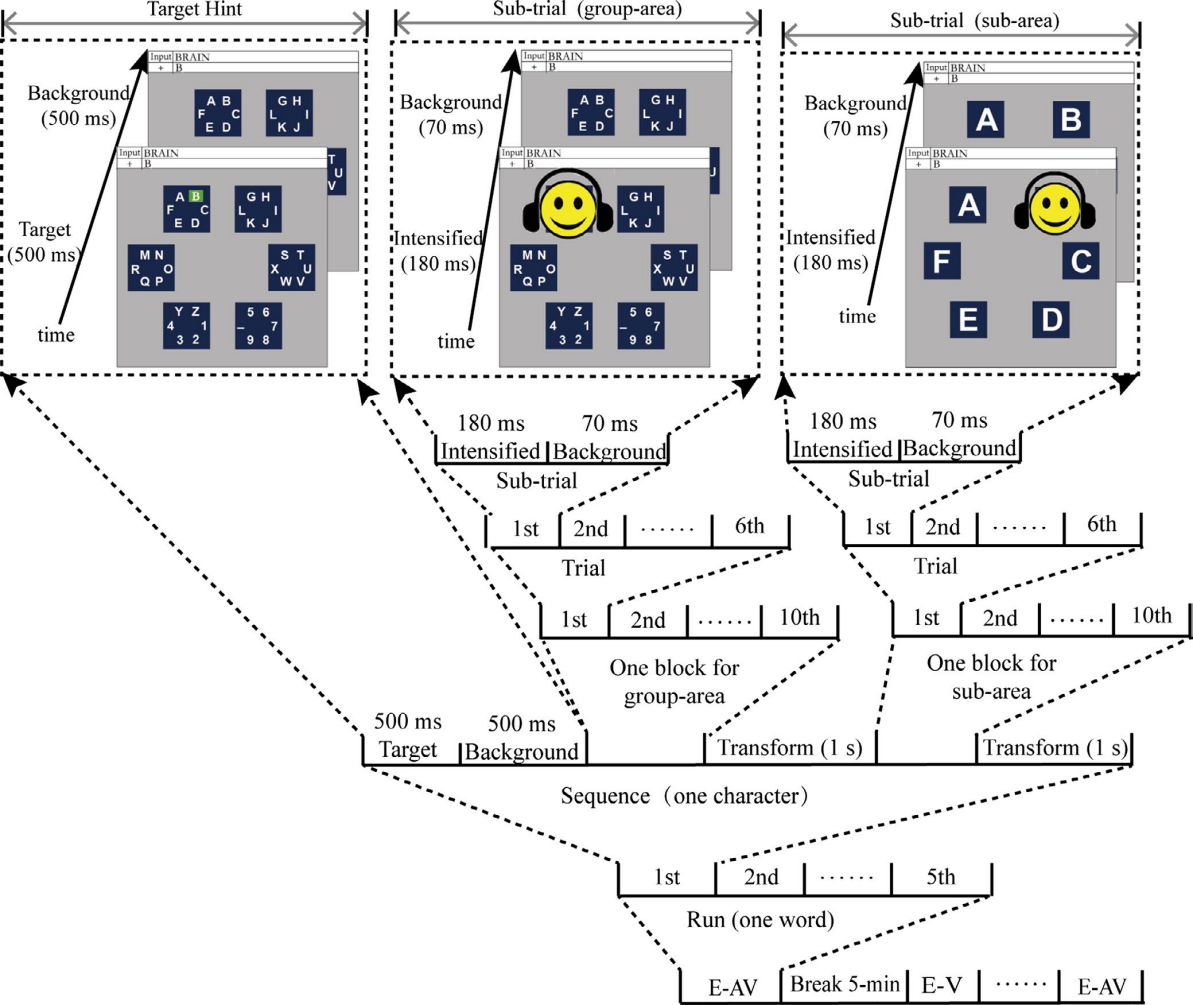
Time course of the experiment

The experiment was conducted in an acoustically shielded room with dimmed lights. Subjects were seated in a comfortable chair and put their chin on a chin rest to keep their eyes at a distance of approximately 70 cm from the computer monitor. Subjects were familiar with the experimental stimuli (visual and auditory) and the task (i.e., silently counting the number of times the target stimulus was presented) and were instructed to keep eye movements or any other body movements to a minimum during the stimulus presentation. During the experiment, each subject performed both spelling paradigms (E‐AV and E‐V) and each paradigm was repeated five times, with subjects spelling one word with five different characters each time (i.e., each subject spelled ten words). The process of spelling one word was as follows: the first target character in the word was first presented on a green background for 500 ms, and then, the screen reverted to the background for 500 ms (Figure 2). The six group‐areas in level 1 were then flashed in pseudorandom order (one flash of a group‐area was defined as a subtrial, and a trial consisted of each of the six group‐areas flashing once, which also referred a superposition; see Figure 2). After each group‐area had flashed 10 times (i.e., after 10 trials, defined as a block; see Figure 2), the screen reverted to the background of level 1 for 1 s, and then, the experiment moved on to level 2, indicated by a “spread” of the group‐area containing the target character. Similarly, the six subareas in level 2 flashed 10 times in pseudorandom order (one block), and then, the screen returned to the background of level 2 for 1 s to indicate the end of the spelling of the first character. The spelling of one character thus consisted of the presentation of the target character, one group‐area block, and one subarea block, all representing one sequence (Figure 2). Next, the second target character was presented and the process was repeated until all five characters of a word were spelled. After each word, subjects took a 5‐min break. The 10 words for the two paradigms were spelled in pseudorandom order, to avoid learning effects.

### 2.4 Data acquisition and preprocessing

**Figure 3:**
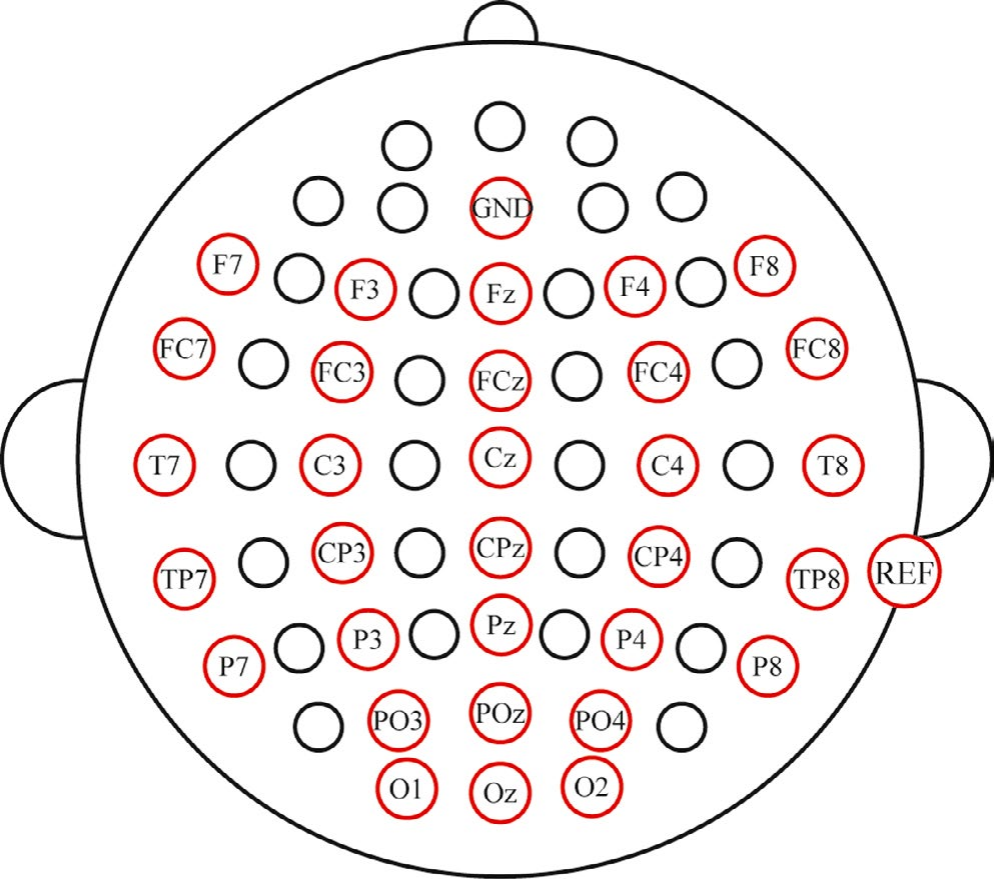
Electrode locations and configuration. “GND” presents ground electrode, and “REF” presents reference electrode

We selected 31 Ag/AgCl scalp electrodes (shown in Figure 3) to record EEG data using the NeuroScan amplifier (SynAmps 2, NeuroScan Inc.). All electrodes were referenced to the right mastoid and grounded to the AFz; impedance was kept below 5 KΩ for all subjects. A pair of vertical electrooculography electrodes was used to record vertical eye movements and eye blinks, and a pair of horizontal electrooculography electrodes was used to detect horizontal eye movements. EEG signals were band‐pass filtered at 0.1–100 Hz, and the sampling frequency was 250 Hz. All experimental paradigms were implemented using E‐prime 2.0 software (PST Inc.). The preprocessing of EEG data for subsequent analyses and offline classification was conducted using Scan4.5 software (NeuroScan Inc).

Electroencephalography data preprocessing included ocular correction using a regression analysis algorithm (Semlitsch, Anderer, Schuster, & Presslich, 1986), division of each subtrial to epochs (−100 to 800 ms), baseline correction (−100 to 0 ms), and removal of bad trials (±80 μV). Subsequently, the averaged ERP data for target and nontarget trials were used for ERP waveform analysis and the digitally filtered EEG data (using a third‐order Butterworth band‐pass filter of 0.01–30 Hz) were used for feature extraction and classification.

### 2.5 Feature extraction and classification

Feature extraction for classification was based on the temporal and spatial features of the EEG data. For the temporal and spatial features, the selection of a time window and electrode with obvious ERP components elicited by the target stimuli and ERP amplitudes with significant differences between target and nontarget stimuli is helpful for classification accuracy. The *r* ^2^ values can provide the mathematic foundation for electrode and time window selection (Cao et al., 2017). The *r* ^2^ was calculated using the following formula:

(1) $$r^{2}={\left(\frac{\sqrt{N_{1}N_{2}}\left(\text{mean}\;\left(x_{1}\right)-\text{mean}\;\left(x_{2}\right)\right)}{\left(N_{1}+N_{2}\right)\text{std}\left(x_{1}\cup x_{2}\right)}\right)}^{2}$$

where *N*_1_ and *N*_2_ represent the sample sizes of the target and nontarget, respectively, and *x*_1_ and *x*_2_ are feature vectors of the target and nontarget, respectively.

The EEG was then down‐sampled from 250 to 50 Hz by selecting every five samples from the epoch. Thus, the size of the feature vector was *C_N_* × *P_N_* (*C_N_* represents the number of channels, and *P_N_* represents the sample points).

Next, we employed Bayesian linear discriminant analysis (BLDA), a classical binary classification algorithm that has been widely used for the classification of EEG signals in BCI systems, which applies regularization to prevent overfitting in high dimensional and possibly noisy datasets (Hoffmann, Vesin, Ebrahimi, & Diserens, 2008). In this study, four of the five words in each spelling paradigm were classified as training data and the remaining word was classified as test data. Alternating each of the five words as test data, we calculated the average accuracy and, finally, the spelling accuracy of each subject.

### 2.6 Raw bit rate

In this study, we used a bit rate calculation method, raw bit rate (RBR), to facilitate comparisons with other studies. The bit rate is an objective measure of the BCI performance that can be used for comparing different BCIs (Wolpaw et al., 2002). The RBR was defined in the study of Wolpaw et al. (2002). In our study, it was calculated without selection time.

### 2.7 Statistical analysis

Based on the ERP components elicited by the target stimuli in the present two paradigms and previous findings, we compared and analyzed the mean amplitudes of P200 (180–220 ms, Cui, Ma, & Luo, 2016), N200 (180–220 ms, Jachmann, Drenhaus, Staudte, & Crocker, 2019), P300 (240–460 ms, Cui, Ma, et al., 2016; Zhu et al., 2019), N400 (340–440 ms, McDermott & Egwuatu, 2019), and P600 (500–640 ms, Cui, Ma, et al., 2016) between E‐V and E‐AV target stimuli. Referencing previous studies that the obvious P200, N200, P300, N400, and P600 were observed at the electrodes' location, data analysis was performed using the F3, Fz, F4, FC3, FCz, FC4, C3, Cz, and C4 electrodes for the P200 components (Cui, Ma, et al., 2016); P3, Pz, P4, PO3, POz, and PO4 electrodes for the P300 components (Cui, Ma, et al., 2016; Zhu et al., 2019); P7, P3, Pz, P4, and P8 electrodes for the N200 components (Jachmann et al., 2019); F7, F3, Fz, F4, F8, FC3, FCz, FC4, C3, Cz, and C4 electrodes for the P600 components (Cui, Ma, et al., 2016; Speer & Curran, 2007); and the F7, F3, Fz, F4, F8, FC3, FCz, and FC4 electrodes for the N400 components (Jachmann et al., 2019). One‐way repeated measures analysis of variance (ANOVA) was performed with the two within‐subject factors, the spelling paradigm (E‐V, E‐AV) and electrode positions, followed by post hoc comparisons performed with Bonferroni correction for the significant results (Zhu et al., 2019). For statistical comparison of the accuracy and RBR at each superposition (the number of times trials were repeated) between the two spelling paradigms, we used the pairwise *t* tests. All statistical analyses were conducted using the SPSS version 19.0 software package (IBM Corp.).

## 3 RESULTS

### 3.1 ERP results

**Figure 4:**
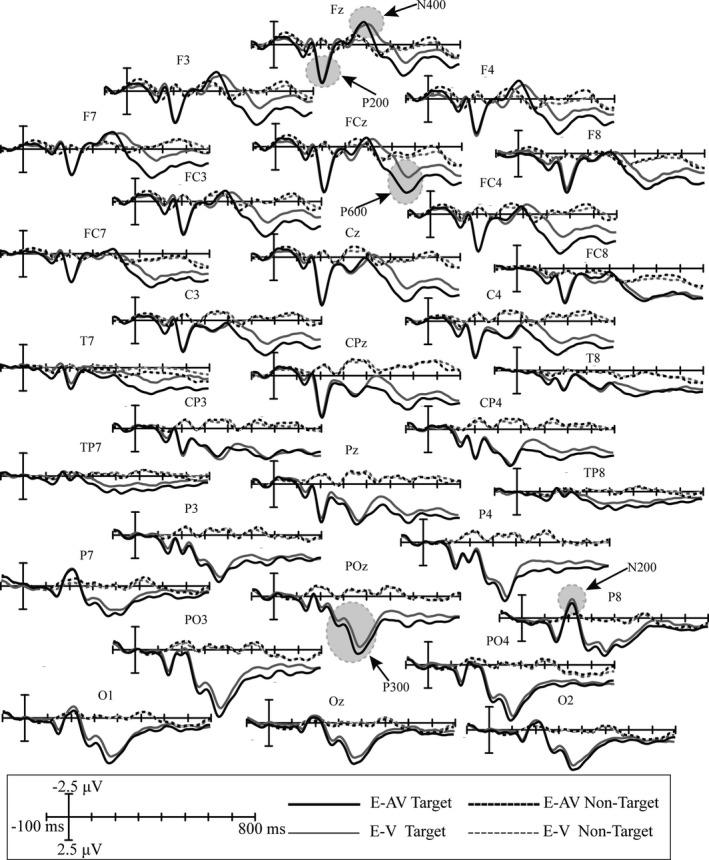
Superimposed grand‐averaged ERPs elicited by target and nontarget stimuli over all 31 electrodes in the E‐V and E‐AV spelling paradigms. E‐AV Target: the waveform elicited by the audiovisual target stimuli; E‐AV Nontarget: the waveform elicited by the audiovisual standard stimuli; E‐V Target: the waveform elicited by the visual target stimuli; E‐V Nontarget: the waveform elicited by the visual standard stimuli

The ground average waveforms of targets and nontargets across all subjects over the 31 electrodes in the E‐V and E‐AV spelling paradigms are shown in Figure 4. The figure shows several obvious ERP components elicited by the target stimuli in both paradigms.

**Figure 5:**
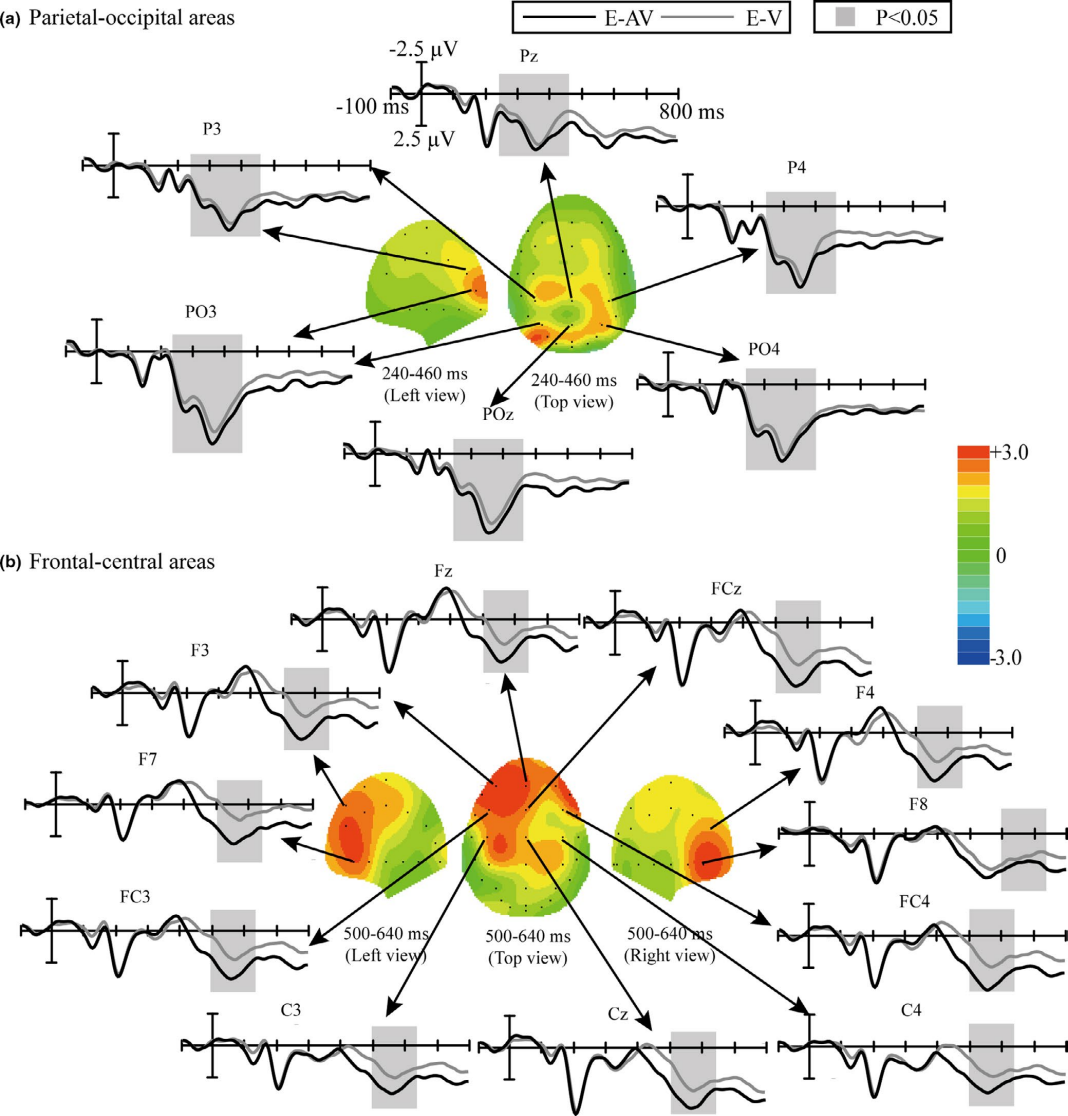
Comparison of waveforms elicited by the target trials in the E‐V and E‐AV spelling paradigms and scalp topographies from the difference in waveforms obtained by subtracting the ERPs of the E‐V spelling paradigm from those of the E‐AV spelling paradigm. (a) Parieto‐occipital areas at 240–460 ms; (b) frontal–central areas at 500–640 ms

The first ERP waveform with a positive peak was observed at the frontal and central channels at approximately 200 ms; this was identified as the P200 (Figure 4). The statistical analysis showed that the main effects of spelling paradigm and electrode position for the P200 amplitude were not significant (spelling paradigm (*F* [1, 18] = 3.392, *p* > .05); electrodes position (*F* [1, 18] = 2.168, *p* > .05)). The second obvious positive ERP waveform was seen at the parietal channels, with two peaks at approximately 280 and 360 ms (Figure 4), identified as the P300. The statistical analysis showed that the main effect of spelling paradigm for the P300 amplitude was significant (*F* [1, 18] = 6.501, *p* = .021 < 0.05, $\eta_{p}^{2}$ = 0.277; Figure 5a), but the main effect of electrode position was not significant (*F* [1, 18] = 1.498, *p* > .05). The interaction effect of spelling paradigm and electrode position for the P300 amplitude was also not significant (*F* [1, 18] = 0.938, *p* > .05). Bonferroni post hoc analysis was conducted based on the ANOVA analysis of the P300 amplitude, and the results showed that P300 amplitudes were significantly larger in the E‐AV spelling paradigm than those in the E‐V spelling paradigm at the P3, Pz, P4, PO3, POz, and PO4 electrodes (*p* < .05).

The third high positive ERP waveform was observed at the frontal and central channels between 500 and 640 ms (Figure 4), considered to be the P600. The statistical analysis showed that the main effect of spelling paradigm for the P600 amplitude was significant (*F* [1, 18] = 6.025, *p* = .025 < 0.05, $\eta_{p}^{2}$ = 0.262; Figure 5b), but the main effect of electrode position was not significant (*F* [1, 18] = 1.035, *p* > .05). The interaction effect of spelling paradigm and electrode position for the P600 amplitude was not significant (*F* [1, 18] = 0.808, *p* > .05). Bonferroni post hoc analysis showed that the P600 amplitudes were significantly larger in the E‐AV spelling paradigm than those in the E‐V spelling paradigm at the F7, F3, Fz, F4, F8, FC3, FCz, FC4, C3, Cz, and C4 electrodes (*p* < .05).

In addition, there were two obvious negative ERP waveforms. One was at the frontal channels with a peak at approximately 400 ms (Figure 4), presumably the N400. The statistical analysis showed that the main effects of spelling paradigm and electrode position for the N400 amplitude were not significant (spelling paradigm (*F* [1, 18] = 4.201, *p* > .05); electrode position (*F* [1, 18] = 2.311, *p* > .05)). The other negative ERP waveform was with a peak at approximately 200 ms (Figure 4), identified as the N200. The statistical analysis showed that the main effects of spelling paradigm and electrode position for the N200 amplitude were not significant (spelling paradigm (*F* [1, 18] = 2.159, *p* > .05); electrode position (*F* [1, 18] = 2.683, *p* > .05)).

Figure 5 depicts the scalp topographies corresponding to the ERP waveforms with significant difference obtained by subtracting the waveforms elicited by the target stimuli in the E‐V spelling paradigm from those elicited in the E‐AV spelling paradigm.

**Figure 6:**
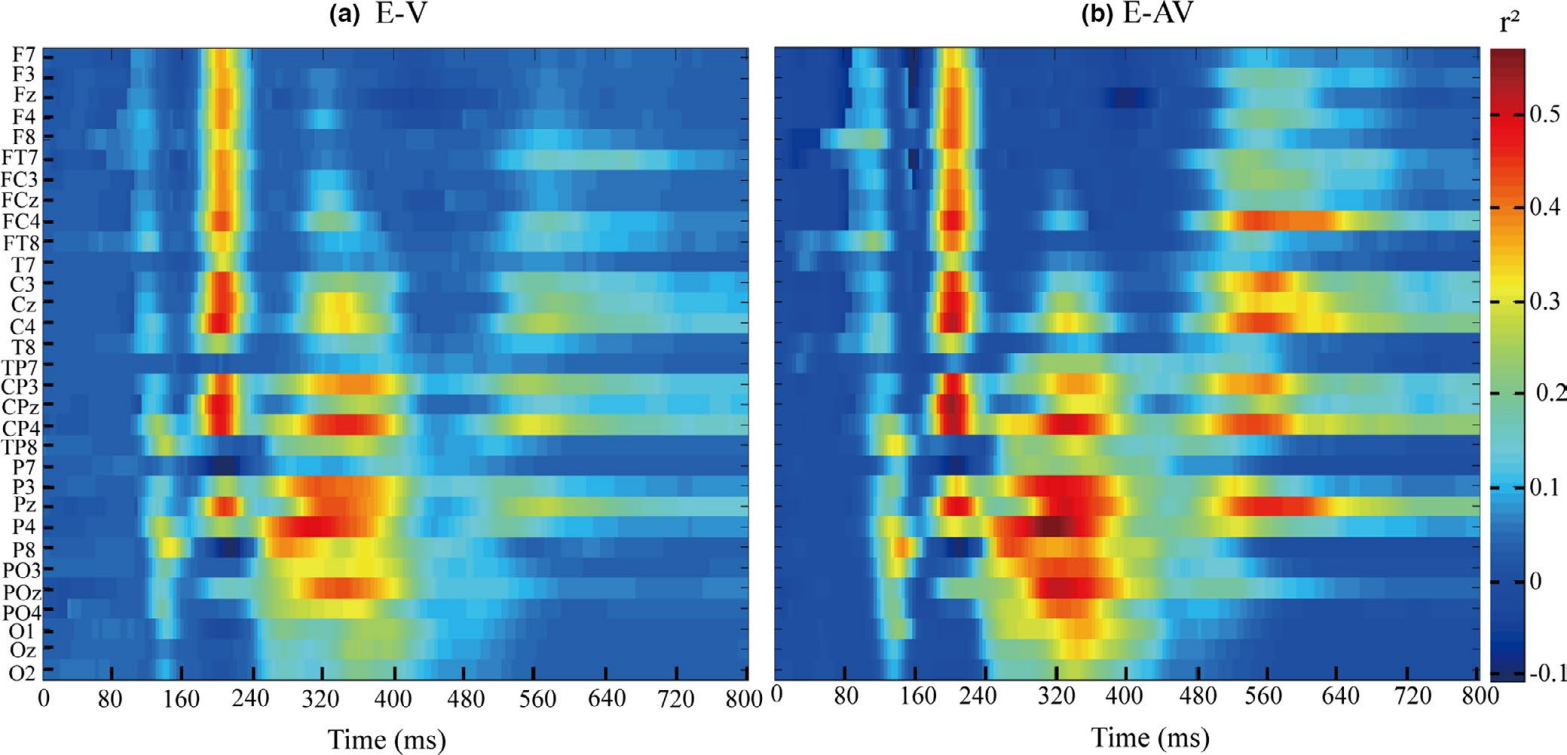
*R*‐squared values of ERP amplitudes elicited by target and nontarget stimuli at 0–800 ms based on the EEG data of all subjects in the E‐AV and E‐V spelling paradigms. (a) *R*‐squared values of ERPs for the E‐V spelling paradigm. (b) *R*‐squared values of ERPs for the E‐AV spelling paradigm

The feature differences of the EEG data between the target and nontarget stimuli in the E‐V and E‐AV spelling paradigms were indicated by the *r* ^2^ values (Figure 6). The differences of the temporal and spatial features between the target and nontarget stimuli were mainly between 200 and 240 ms at the F7, F3, Fz, F4, FT7, FC3, FCz, FC4, C3, Cz, C4, CP3, CPz, CP4, and Pz electrodes and between 240 and 440 ms at the CP3, CP4, P3, Pz, P4, and POz electrodes in the E‐V spelling paradigm, whereas for the E‐AV spelling paradigm, the feature differences were observed between 200 and 240 ms at the F7, F3, Fz, F4, F8, FT7, FC3, FCz, FC4, FT8, C3, Cz, C4, CP3, CPz, CP4, and Pz electrodes, between 240 and 440 ms at the CP3, CP4, P3, Pz, P4, P8, PO3, POz, and PO4 electrodes, and between 480 and 640 ms at the FC4, C3, Cz, C4, CP3, CP4, and Pz electrodes. In addition, the feature difference between 240 and 400 ms in the E‐AV spelling paradigm was larger than that in the E‐V spelling paradigm.

### 3.2 Classification accuracy and RBR

**Figure 7:**
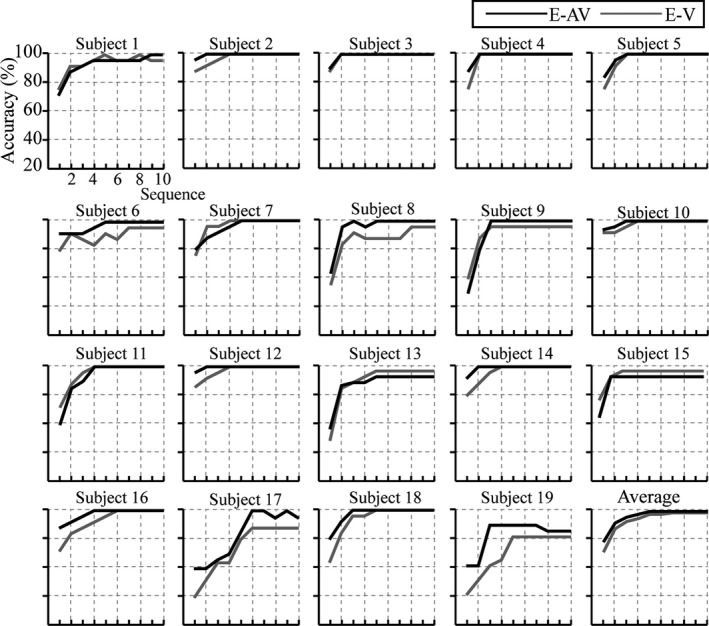
Individual and average accuracies of the P300 E‐V and E‐AV spelling paradigms for the 19 subjects

Based on the ERP analysis and comparison of the *r* ^2^ values, the feature vector used to classifying was set as 30 × 24 (30 represents the sample points between 100 and 700 ms; 24 represents channels F7, F3, Fz, F4, F8, FT7, FC3, FCz, FC4, FT8, C3, Cz, C4, CP3, CPz, CP4, P7, P3, Pz, P4, P8, PO3, POz, and PO4). Figure 7 shows the offline classification accuracy of the E‐V and E‐AV spelling paradigms at each superposition. The accuracy increased with the number of superpositions in both the E‐V and E‐AV spelling paradigm, reaching 100% at only two superpositions (subjects 2, 3, 4, 12, and 14 in the E‐AV spelling paradigm and subject 3 in the E‐V spelling paradigm). The average superposition time was 3.2 when accuracy reached 100% for thirteen subjects in the E‐AV spelling paradigm and 3.9 when accuracy reached 100% for eleven subjects in the E‐V spelling paradigm.

We conducted *t* tests to compare the accuracy at each superposition time and found significant differences between the two spelling paradigms at one superposition ([E‐V, E‐AV], *t* = −2.642, *p* = .017 < 0.05) and two superpositions ([E‐V, E‐AV], *t* = −2.242, *p* = .038 < 0.05).

**Figure 8:**
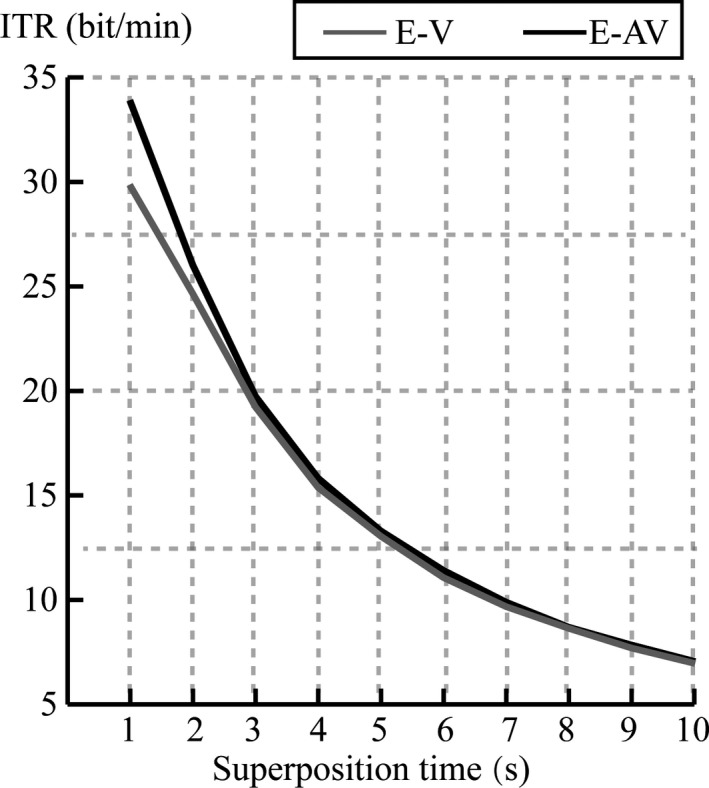
Average RBR at each superposition time for the E‐V and E‐AV spelling paradigms

The average RBR at each superposition time for the 19 subjects in the E‐AV and E‐V spelling paradigms is shown in Figure 8. The RBR was greater in the E‐AV spelling paradigm than that in the E‐V spelling paradigm at superposing one, two, three, and four times. The result of the *t* test showed that there was a significant difference in the RBR between the E‐AV and E‐V spelling paradigms at one superposition ([E‐V, E‐AV], *t* = −3.046, *p* = .007 < 0.05) and two superpositions ([E‐V, E‐AV], *t* = −2.154, *p* = .045 < 0.05).

## 4 DISCUSSION

In this study, we designed a new audiovisual P300 speller system based on the congruence of happy emotions in faces and voices to verify whether bimodal emotional face and voice stimuli can further improve the spelling accuracy of such a system, compared with visual‐only face stimuli. We assessed the validity of the hypothesis by analyzing the ERPs elicited by the target stimuli and comparing the spelling accuracy between the bimodal (E‐AV) and unimodal (E‐V) paradigms.

### 4.1 ERP analysis

The visual‐only smiling face stimuli and the audiovisual stimuli consisting of a smiling face and a chuckle both elicited obvious ERP components, such as P200, N200, P300, N400, and a positively deflected waveform between 500 and 640 ms (P600; Figure 4). However, the audiovisual stimuli elicited two ERPs with significantly larger amplitudes than those elicited by the visual‐only stimuli.

The first ERP whose amplitude showed a significant difference between the E‐AV and E‐V spelling paradigms, the P300, occurred at the parieto‐occipital area from 240 to 460 ms (Figure 5). The P300 is a positive component and has more parietal distributions that occur between 200 and 500 ms after stimulus onset, related to attention and cognitive processing (Polich, 2007). In addition, the P300 has been reported to be associated with attentive processing of both facial and vocal emotion (Campanella et al., 2013; Paulmann, Jessen, & Kotz, 2012). In a study on bimodal emotion integration, P300 amplitudes were larger for audiovisual emotion stimuli than for visual emotion stimuli; the authors suggested that the bimodal stimuli led to a “dual novelty” in the cognitive task comprising visual and auditory stimuli, which enabled subjects to actively process multisensory information (Chen, Han, et al., 2016). Similar findings were also reported in some studies on the sensitivity of P300 to emotional face–voice stimuli (Campanella et al., 2010), the integration of facial and vocal emotion perception (Chen, Pan, et al., 2016), and emotion recognition tasks (Liu et al., 2012). In addition, changes in age and sex features in voices have also been shown to increase subjects' attention to and perception of stimuli (Li et al., 2015). This explains why, in our study, the smiling face and chuckle stimuli in the E‐AV spelling paradigm elicited larger P300 amplitudes than those elicited by the smiling face stimuli in the E‐V paradigm; subjects presumably used more attentional resources on the audiovisual stimuli and processed the emotional information in these stimuli more actively.

The second ERP amplitude that showed significant differences between the two spelling paradigms appeared at the frontal–central areas from 500 to 640 ms and is presumably the P600 (Figure 5). Some studies suggested that the P600, with a more frontal–central distribution between 500 and 600 ms, is associated with recollection (Duarte, Ranganath, Winward, Hayward, & Knight, 2004; MacKenzie & Donaldson, 2007; Speer & Curran, 2007). One study on emotional source memories found that the P600 amplitude increased when subjects showed an enhanced memory bias for emotion information related to source familiarity (Cui, Shi, et al., 2016). In our study, we selected six smiling face and chuckle pairs from male and female individuals of three different age groups. When the six bimodal pairs or the six smiling faces were presented in random order, we observed a memory bias not only for visual emotion, but also for auditory emotion, for the audiovisual stimuli relative to the visual‐only stimuli, which presumably enhanced subjects' recollection and resulted in the P600 amplitude increase in the E‐AV paradigm compared with that in the E‐V paradigm.

### 4.2 Spelling accuracy and RBR

Spelling accuracy is an important index to measure the performance of a P300 speller system. In particular, the spelling accuracy was on a lesser number of superposition times for stimuli that could improve the RBR. Because the accuracy appeared stable after four superpositions in 16 subjects in the E‐AV spelling paradigm, we compared the accuracy between the two paradigms at the first four superpositions. We found that the mean accuracies in the E‐AV spelling paradigm were higher than those in the E‐V spelling paradigm at the first four superpositions. The E‐AV spelling paradigm significantly improved the accuracy at the first two superpositions (*p* < .05) compared with those in the E‐V spelling paradigm. These results verify that bimodal emotion stimuli elicit larger‐amplitude ERPs than unimodal emotion stimuli, which can thus improve the accuracy of the P300 speller system. In addition, the RBR is also an important statistical metric for BCI systems (McFarland, Sarnacki, & Wolpaw, 2003); it comprehensively evaluates the accuracy and output speed of character spelling. In our study, the RBRs were significantly higher in the E‐AV spelling paradigm than those in the E‐V spelling paradigm at one and two superposition times (*p* < .05), which indicated that the E‐AV spelling paradigm significantly improved the performance of the P300 speller compared with the E‐V spelling paradigm.

### 4.3 Potential advantage for users

This P300 speller system represents a type of cross‐modal audiovisual BCI system, which offers two main potential advantages to users. First, the visual and auditory stimuli in the cross‐modal system complement each other; thus, when users are distracted or tired, the bimodal stimuli lead to more robust results than do unimodal stimuli. For example, when users cannot visually distinguish how many times the target flashes, they can rely on the auditory information. Second, this bimodal P300 BCI system can be directly converted to a unimodal BCI system for users with visual or auditory degradation or loss due to a disease.

### 4.4 Performance comparison of state‐of‐art P300 speller based on emotional cognition

**Figure 9:**
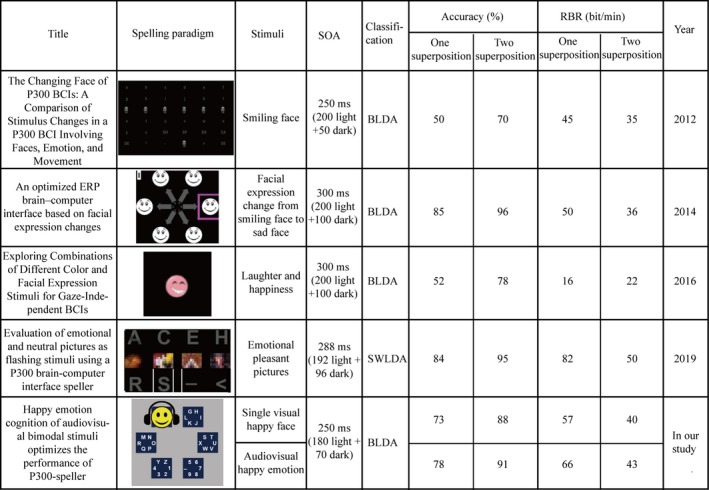
Performance comparison of state‐of‐art P300 spellers based on emotional cognition

Since the control paradigm in the present study was the visual emotion‐based spelling paradigm, we searched all visual emotional spelling paradigms from PubMed for the effectiveness of comparison and compared the performances of these P300 spellers in terms of the design of the stimulus paradigm, stimuli onset asynchrony (SOA), classification algorithm, spelling accuracy, and RBR. The details of the comparison are shown in Figure 9. In our study, the accuracy and RBR were significantly greater in the E‐AV spelling paradigm than those in the E‐V spelling paradigm at superposing one and two times; thus, the comparison of accuracy and RBR between the spelling paradigms was at superposing one and two times. From the Figure 9, we observed that the accuracy of the E‐AV was higher than that in 2012 (Jin et al., 2012) and 2016 (Chen, Pan, et al., 2016) at one and two superpositions and the RBR of the E‐AV was higher than that in the 2012, 2014 (Jin et al., 2014), and 2016 at one and two superpositions. The accuracy of the spelling paradigm is affected by several factors, such as the arrangement of the characters, visual angle (subjects spelled more accurately on a larger visual angle than on a smaller visual angle (Li, Nam, Shadden, & Johnson, 2011), SOA (increased SOA would result in a larger P300 amplitude to improve the classification accuracy [Lu, Speier, Hu, & Pouratian, 2013]), and other factors. The visual angles of the spelling matrix in 2019 (Fernandez‐Rodriguez, Velasco‐Alvarez, Medina‐Julia, & Ron‐Angevin, 2019) with 16.31° × 23.54° and in 2014 with 19.7° × 32.7° were greater than that in our experiment (13.4° × 19.4°), and the SOAs in 2014 (300 ms) and in 2019 (288 ms) were greater than that in our experiment (250 ms). These are probably the reasons why the accuracy of their spelling paradigm is greater than that of our spelling paradigm. In addition, the RBR depends on both classification accuracy and character output speed, and the speed of character output depends on the length of SOA. Therefore, the higher RBR was obtained need to weight the classification accuracy and the speed of character output. In the 2019 study, the set of SOA achieved a higher accuracy without weakening the RBR, which may be the reason why the RBR of their spelling paradigm was greater than that of our spelling paradigm. In future, we need to determine how to adjust the SOA to improve the RBR while ensuring stability of accuracy to further optimize the performance of the audiovisual P300 speller.

### 4.5 Limitations and future work

The design of this audiovisual P300 spelling paradigm was based on a traditional region flashing paradigm that contains two levels, character group‐areas and single‐character subareas. The output of one character was created by locating the group‐area and subarea containing the target character, which means that the setting of the conversion time between the group‐area and subarea may have affected the spelling speed of the system. Other factors, such as the interstimulus interval or the appropriate repetition times of the stimuli, may also affect its performance. Further work is therefore needed to specify these parameters and thereby optimize the performance of the audiovisual P300 speller system.

## 5 CONCLUSION

This study presents an audiovisual P300 speller system based on the congruence of happy emotions in faces and voices. Compared with the emotion‐based visual‐only P300 speller system, this system demonstrated significantly improved character spelling accuracy at the first two superpositions. In addition, this P300 speller enhances the universality of the system, because it can be adapted to any unimodal auditory or visual spelling system according to the user's needs.

## CONFLICT OF INTEREST

The authors declare that the study was conducted in the absence of any commercial or financial relationships that could be construed as a potential conflict of interest.

## Data Availability

The data that support the findings of this study are available from the corresponding author upon reasonable request.
